# Comparative Outcomes Assessment: Hip Hemiarthroplasty as an Alternative to THA in Patients with Surgically Pristine Acetabulum—Is There Still a Role?

**DOI:** 10.1155/2013/632126

**Published:** 2013-08-12

**Authors:** Thomas B. Pace, Brad Prather, Brian Burnikel, Brayton Shirley, Stephanie Tanner, Rebecca Snider

**Affiliations:** ^1^Greenville Health System Department of Orthopaedics, 701 Grove Road, Greenville, SC 29607, USA; ^2^GHS, Orthopaedic Surgery, University of South Carolina SOM, Greenville Health System, P.O. Box 27114, Greenville, SC 29616, USA; ^3^Hendricks Orthopedics and Sports Medicine, 300 Hospital Lane Suite 300, Danville, IN 46122, USA; ^4^Steadman Hawkins Clinic of the Carolinas, 200 Patewood Drive, Suite C100, Greenville, SC 29615, USA

## Abstract

This is a retrospective review of 243 hip arthroplasties treated with either hemiarthroplasty (61 surgeries-Group 1) or total hip arthroplasty (182 surgeries-Group 2). The mid- to long-term results of relatively similar, predominately young patient cohorts were assessed annually via radiographs and the Harris Hip Scores for pain and clinical function. Groin pain persisted in 16.4% of Group 1 and 5.5% of Group 2 (*P* = 0.0159). Thigh pain persisted in 11.5% of Group 1 and 2.2% of Group 2 (*P* = 0.0078). Complications in Group 1 were 4/61 including 2 revisions with an overall survival rate of 96.7% versus Group 2 complication rate of 29/182 with 15 revisions and an overall survival rate of 91.8%. There were no cases of acetabular protrusio in Group 1, but 2 cases (1%) in Group 2 had cup loosening or osteolysis. Two cases were revised in Group 1 (3.2%). Both were undersized femoral stems. The fifteen revisions (8.2%) in Group 2 included loose stem (1), instability (8), infections (3), cup loosening (2), and accelerated polyethylene wear (1). Hemiarthroplasty has a higher incidence of thigh and groin pain but fewer complications compared with total hip arthroplasty.

## 1. Introduction

The use of hemiarthroplasty was initially advocated for femoral neck fractures. Hemiarthroplasty advocates indicate ease of implantation, reduced blood loss, a lower dislocation rate, and the ease of acetabular revision when compared with conventional total hip arthroplasty (THA) [1–3]. Historically, surgeons have advocated the use of hemiarthroplasty for the treatment of degenerative arthritis, fracture, or avascular necrosis (AVN) of the hip based primarily on relatively young patient age and the benefit of preserving the acetabular bone stock for future anticipated surgeries. With the availability of newer technologies such as hard on hard bearings (metal on metal, ceramic on ceramic), highly cross-linked polyethylene, and resurfacing arthroplasties, one may consider whether hemiarthroplasty in younger patients is an operation of the past.

Many patients needing hip arthroplasty suffer from hip osteonecrosis, often historically referred to as AVN, of the femoral head [4]. The clinical diagnosis of osteonecrosis is often a general one associated with multiple other conditions and with many features of hip osteoarthritis (OA) in its late stages, thus making large patient series difficult to accumulate and even more difficult to compare. Studies such as Wroblewski et al. had a series of 49 patients identified with eleven distinct diagnoses within AVN [5]. There are some longer-term studies of implant use with a diagnosis of AVN [2, 3, 6, 7]. While conclusions in these studies mostly reflect lower failure rates and improved symptomatic results with THA, both conventional THA and bipolar arthroplasty have been successful. 

In younger and smaller patients, in an effort to maximize the polyethylene within the hemiarthroplasty cup to increase longevity, the acetabular cartilage and subchondral bone were often overreamed surgically to allow a larger articulating bipolar head. The historical literature suggests a higher rate of revision surgery in hemiarthroplasties primarily due to groin pain, progressive arthritis, and acetabular protrusion, while standard THA has a comparably higher rate of dislocations [8]. Hemiarthroplasty used in a primary setting has a minimal incidence of dislocation [9]. For reasons not entirely clear, it has been reported that the revision or failure rate of THA is up to four times higher when comparing AVN to osteoarthritis [10]. Hemiarthroplasty on the other hand has not been shown to have such a differing result based on etiology of the hip condition [5, 6, 9]. The literature supports long-term diminishing results with hip hemiarthroplasty as a rule but does not discern those with moderately diseased acetabulum from those who have minimally involved acetabular cartilage and well-preserved congruency nor does it differentiate surgical techniques of overreaming the acetabular subchondral bone and replacing it with a fixed arthroplasty cup versus assessing and preserving a pristine native acetabulum and its role in forming a congruent structural framework for a hemiarthroplasty.

There is currently no consensus regarding the best arthroplasty for younger patients when the pathology appears to be limited involvement of the femoral head. In addressing hip osteonecrosis, the majority of surgeons appear to base surgical choices on the staging of the disease which has not been correlated with outcome [9, 10]. The purpose of this study is to compare the results of hemiarthroplasty with total hip arthroplasty for selective patients with well-preserved acetabulum. 

## 2. Materials and Methods

This institutional review board-approved review involved a retrospective analysis of demographic, radiographic, and standardized outcome data concerning a cohort of 366 patients similar in age and gender receiving one of two types of hip arthroplasty. 

Group 1 (hemiarthroplasty group) consisted of patients with one of three presurgical hip conditions: (a) early but painful hip osteoarthritis limited to focal chondrolysis of the femoral head, (b) early (Ficat stages II and III) hip osteonecrosis without MRI or visual evidence of acetabular involvement at the time of surgery, or (c) femoral neck fractures with pristine acetabular cartilage. These patients were selected at the time of surgery to receive a hemiarthroplasty (all bipolar implants) rather than a fixed cup (THA) based on the findings of a pristine acetabular articular cartilage surface and “suction fit” cup of a trial implant. The tight “suction fit” of the hemiarthroplasty implant trial at the time of surgery was felt to indicate well-preserved and congruous acetabular cartilage and subchondral acetabular bone. If a trial cup implant suction fit was not tight enough to “shift the pelvis,” a hemiarthroplasty was not performed; the acetabulum was then prepared and THA performed.

The hemiarthroplasty cohort included patients meeting the inclusion criteria with surgery performed between 1995 and 2007 accounting for 115 cases in 103 patients. Forty-five patients were excluded due to inadequate followup and 8 patients were lost due to death unrelated to the prosthesis or arthroplasty procedure. These exclusions leave a cohort of 50 patients having 61 procedures for comparison. Diagnoses by procedure included 40 with AVN (Ficat stages II and III, no stage four—acetabular involvement), 9 fractures, and 12 patients with osteoarthritis visibly limited to focal involvement of the femoral head at the time of surgery. The average patient age was 56.5 years (range 24–90); 61% were females. The average patient followup in this group was (8.2) years (range 2–15.6). 

As a comparative group, Group 2 (THA group) consisted of patients who received conventional THA performed by the same surgeon during a similar time period (1993 to 2001). This initial group included 300 total hip arthroplasty cases in 263 patients. Ninety-five patients were excluded due to inadequate follow-up data and 31 patients were lost due to death. Exclusions reduced the cohort to 156 patients having 182 conventional THA procedures. Diagnoses within the THA group included 141 osteoarthritis, 4 fractures, 31 AVN (Ficat stage IV), 2 dysplastic, 2 with RA, and 2 hip fusions. The average patient age in this group was 59 (range 24–86) and 56% were females. The average followup in the group was 10.6 years (range 2–18.4). 

Implants used in both groups consisted exclusively of the Zimmer Natural Hip press fit stem (Zimmer Inc., Warsaw, Indiana). All femoral heads used in Group 1 were cobalt chrome and 26 mm in diameter. The 26 mm diameter head was chosen to maximize polyethylene thickness in this group of patients especially the ones with a small native acetabulum. The smallest bipolar cup used was a 42 mm outer diameter and this allowed for a 7 mm polyethylene articulating with a 26 mm cobalt chrome head. Within the THA cohort, femoral heads were either 28 or 32 mm in diameter as needed to provide the largest head size possible for stability purposes and still preserve a minimum of 9 mm of polyethylene thickness. All polyethylene was nonhighly crossed-linked. All patients receiving hemiarthroplasty had the same standard Cobalt Chrome shell design (Natural Hip, Zimmer Inc., Warsaw, Indiana) and the average size was 48 mm (range 42–58 mm). Patients receiving THA received an intra-op press-fit cup (Zimmer Inc., Warsaw, Indiana). The average cup size was 53.7 mm (range 43–65 mm) and screws were used in 42 THA cases as needed to secure the cup. 

The surgical approach was posterolateral and involved general or spinal anesthesia. Hip capsular reapproximation was performed at wound closure. Postoperative care included ipsilateral immobilizer for 48 hours for dislocation precaution and weight bearing as tolerated. Contralateral cane or a walker was recommended until the surgical limp subsided. All patients underwent a venous thromboembolism (VTE) risk assessment and received warfarin 5 mg daily in hospital post-op as long as the international normalized ratio (INR) was less than 1.5 (prothrombin time ≤ 18 sec) and at discharge either fixed-dose warfarin 2 mg per day for 30 days for the standard-risk VTE patients (without INR monitoring unless bleeding issues occurred) or adjusted-dose warfarin with INR monitoring for the higher-risk VTE patients. Pre- and postassessment Harris Hip Scores were recorded for all patients. Clinical and radiographic assessment was done pre-op and at 2 weeks post-op, 3 months, and annually. Follow-up AP and lateral radiographs were assessed independently by a fellowship trained arthroplasty specialist (coauthors B. Burnikel, B. Shirley and B. Prather) who did not perform the surgery.

The data was analyzed using chi-square and fishers exact probability test (*α* = 0.05).

## 3. Results

As seen in Table 1, the groups were similar regarding the relatively young age (average 56 and 59, resp.) and gender though there was a trend towards more females in Group 1 (61% versus 56%) as well as preoperative and postoperative clinical assessment using the Harris Hip Scoring (HHS) method. The preoperative HHS averaged 67.2 (range 58 to 87) for Group 1 and 67.7 (range 25 to 87) for Group 2. Postoperative HHS averaged 98 (range 70 to 100) for Group 1 and 99.3 (range 75 to 100) for Group 2.

There was a significant difference in reported thigh and/or groin pain as shown in Table 2. Groin pain occurred in 10 patients in each cohort (16.4% of Group 1 and 5.5% of Group 2). Thigh pain was noted in 7 Group 1 patients (11.5%) and 4 Group 2 patients (2.2%). Aside from the patients in each group who eventually had revision surgery, the patients in both groups with residual thigh and or groin pain did not require pain medication stronger than over-the-counter anti-inflammatory medicines.

**Figure 1:**
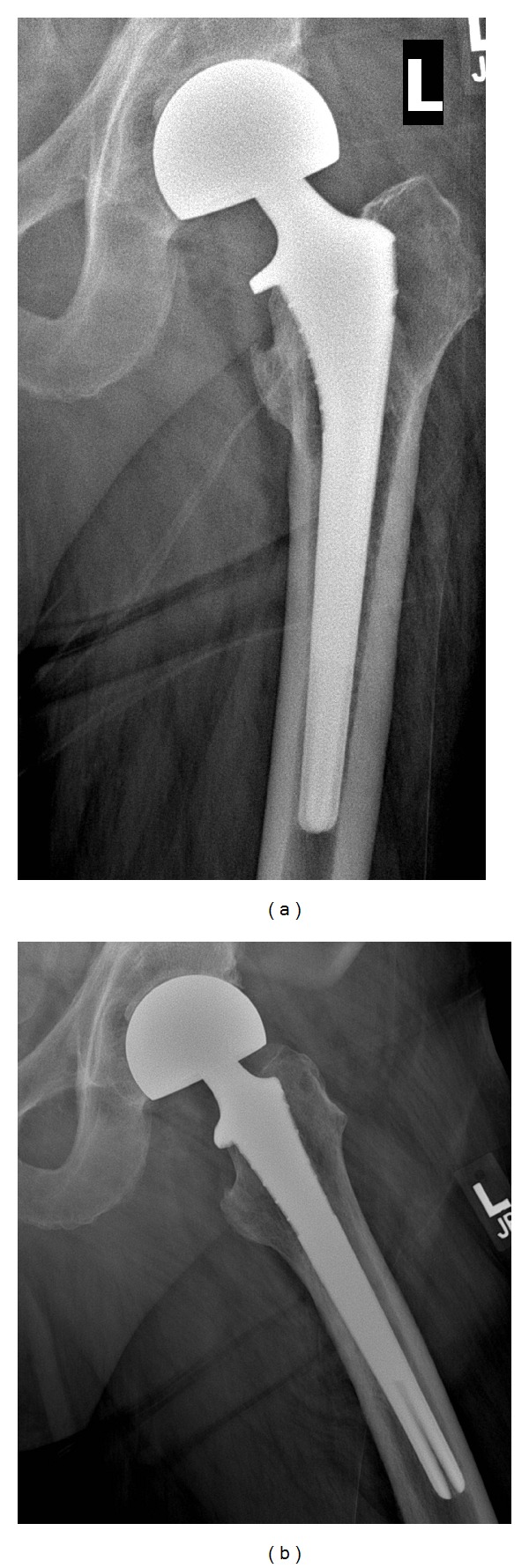
50-year-old construction worker—AP and lateral radiographs—10 years following hemiarthroplasty.

**Figure 2:**
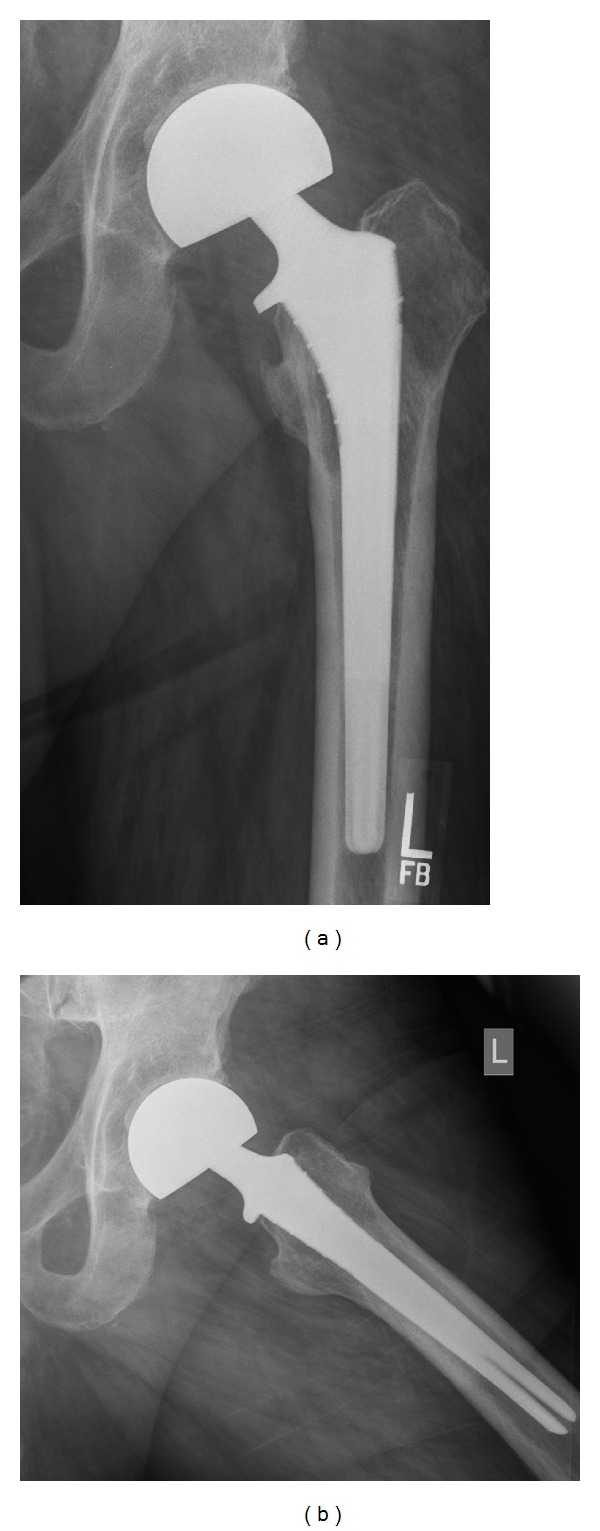
Same patient—AP and lateral radiographs—15 years following hemiarthroplasty.

The results of the independent radiographic assessment are shown in Table 3. Two cups (1.9%) in Group 2 showed cup loosening with migration (one with associated osteolysis) and both eventually required revision surgery. As far out as 15-year followup, no cases of acetabular osteolysis or cup protrusion were found in Group 1 (see Figures 1 and 2). One case of acetabular osteolysis was found in Group 2. Linear polyethylene wear greater than 2 mm was noted in 13 (7%) of Group 2 patients. Polyethylene wear could not to be assessed in Group 1 secondary to the overlying bipolar metal outer cup shell.

On the femoral side, two patients had an undersized stem with subsequent subsidence and revision in Group 1. One patient had an undersized stem in Group 2. All of these patients had previously had hip surgery that deformed the proximal femur (two with hip fracture pins and one with vascularized fibular graft for osteonecrosis). No osteolysis or subcalcar erosion was noted in Group 1. In Group 2, 6 (3%) of the 182 cases had subcalcar erosion of the femur noted on AP and Lateral radiographs. While this was not structurally compromising, it was felt to reflect secondary polyethylene wear osteolysis as it occurred only in the cases of liner polyethylene wear >2 mm and may lead to compromised implant longevity. 

Complications for both groups are shown in Table 4. While there was a trend for more complications in Group 2, it did not reach statistical significance (*P* value = 0.08). Of the patients in Group 1, 4 of the 61 patients (6.5%) had recorded complications. The dislocation rate for the total hip arthroplasty group was significantly higher than the hemiarthroplasty group (*P* = 0.04).

Table 5 shows the reasons for implant revisions for each group. There was a trend towards a higher revision rate in Group 2 (3.2% versus 8.2%) but it did not reach statistical significance (chi square 1.05, *P* value = 0.25). overall implant survival rate was comparable for both groups. The Group 1 survival rate at a mean of 8.2 years was 96.7% versus Group 2 survival rate of 91.8% at a mean of 10.6 years.

## 4. Discussion

There were no revisions in the hemiarthroplasty group for-cup related problems in this series. The two revisions for undersized stems were in patients with prior surgeries with femoral canal deformities. The decreased mild thigh and groin pain in the THA group was significant but at what cost given the higher trend of other complications and revision surgery in this generally younger active patient population. Cabanela and Hanssen et al. compared the use of hemiarthroplasty to THA in patients with osteonecrosis of the femoral head and concluded that a fixed porous-coated acetabular component was associated with better symptomatic improvement and a lower failure rate. In their study, when THA was compared with hemiarthroplasty at a mean of 9.2-year followup, the results of both types of arthroplasty were generally satisfactory [6, 7]. The implants and surgical techniques were completely different between the two groups and the rate of dislocation was higher in the total hip arthroplasty group. This finding has been noted in many other studies as well [11–13]. However, none of the historical reports on hemiarthroplasty outcomes have addressed the issue of assessing the native acetabular condition to essentially allow a suction fit with the new hemiarthroplasty head at the time of surgery. Frequently, the decision to proceed with hemiarthroplasty versus total hip arthroplasty is age based alone and in some reports the acetabulum was actually reamed to accept the hemiarthroplasty head. With the loss of the native acetabular cartilage and supportive subchondral bone, subsequent migration and protrusio of the hemiarthroplasty head in these cases are not surprising.

In a series by Pellegrini et al. good survival of the hemiarthroplasty prosthesis was reported. However of those patients who failed, the acetabular revision more commonly required complex treatment [14]. The hemiarthroplasty implant technique used in that study was to create a concentric acetabulum if one was not present and this is a key differentiating point from the cases presented in this report. It may be that the study by Pellegrini illustrates that manipulation of the acetabulum to accommodate the bipolar head leads to an increased failure rate [14].

In nontraumatic AVN of the hip, the pathology begins in the femoral head and until subchondral collapse occurs the acetabulum may be unaffected structurally. Often replacements occur prior to significant radiographic changes within the acetabulum. However, evaluation of the acetabular bone stock in patients with AVN has shown differing results. Some series reveal that histologic acetabular changes are a minimum when the femoral changes are Ficat stage 1 or 2 [15].

In a histological study by Steinberg et al. evaluation of articular cartilage was performed in patients without radiographic acetabular changes yet revealed that all specimens had histologic changes within the cartilage [16]. They indicated that only one specimen intraoperatively had pristine cartilage, but even this specimen had histologic changes consistent with OA. The Steinberg study did not evaluate the acetabular bone stock but does lend itself to the historical trend of acetabular failure in hemiarthroplasty endoprosthesis as reported by others [9]. One may question whether or not the histologic acetabular changes even in pristine hips translate into structural acetabular deficiencies and subsequent clinical hemiarthroplasty failure. The data reported in this study would suggest that they do not.

Many studies have shown that the short-term retention of components can be good but patients may be symptomatic. The original Bateman hemiarthroplasty prosthetic device success was measured by whether or not the patient could walk at a similar or better than presurgical level. Almost uniformly studies have shown more symptoms in patient receiving hemiarthroplasty. Chan and Shih however demonstrated nearly equivalent results comparing THA with hemiarthroplasty at medium-term followup and from their study proposed hemiarthroplasty as a viable option [9]. Most types of arthritis begin with inflammation of the joint and early degeneration of the cartilage that cannot be detected radiographically. AVN is most likely not different as a degree of degeneration would occur in the articular cartilage of the acetabulum after it had been subjected to a deformed femoral head or to the diseased cartilage of the femoral head [17, 18].

Kindsfater et al. in a study with direct comparison of bilateral hip arthroplasty (THA and hemiarthroplasty in the same patient) reveal similar findings to those longevity studies described thus far. Patients preferred the THA side to the hemiarthroplasty at short- and long-term followup. The case series only involved 9 patients with followup being a limiting factor due to patient death. The series generally indicates THA to be the preferred treatment with a prosthetic survival rate of 95% at eight years [19]. 

The arthroplasty treatment for limited hip disease remains challenging in a younger more active and physically demanding population, as well as patient populations prone to stability and compliance issues secondary to either geriatric senility or substance abuse. There are some who would not suggest hemiarthroplasty as a strong consideration, except for very clearly specified patients based on age and stage of disease [20]. The data from this current report support that recommendation but further define the acceptable conditions to include the surgical findings of a pristine acetabulum and suction fit at the time of surgery rather than patient age as the primary consideration. 

Newer bearing surfaces of hard on hard (metal on metal and ceramic) and highly crossed-linked polyethylene allowing for larger heads with less wear, resurfacing hip arthroplasty all have expanded the options for younger and more demanding patients. These newer technologies of the past decade have proven very encouraging in some aspects and yet have brought some very concerning issues to light in others such as early femoral neck fractures, ion particle concerns, pseudotumors, and ceramic fatigue fracture [21, 22].

There are several limitations to this review that restrict its conclusions and may introduce bias. First, it has a retrospective nature. Secondly, there are somewhat differing patient groups regarding degree of acetabular disease (more advanced in the Group 2), and thirdly, a single surgeon performed both the surgery and subsequent clinical assessment. The strength of the study include the clear distinction of a well-formed preserved acetabulum with a high degree of congruency confirmed at surgery which has not been previously highlighted in prior reports and secondly, the objective independent radiographic assessment by other arthroplasty surgeons blinded to the patient's clinical symptoms strengthens the review and supports the conclusion that the hemiarthroplasty group is not progressing into acetabular protrusio over time. 

## 5. Conclusion

The findings of this study confirm the historically reported higher incidence of thigh and groin pain in the hemiarthroplasty patient group compared with the traditional THA group. Acetabular protrusion of the hemiarthroplasty head was not a clinical or radiographic issue nor was instability or early failure secondary to the acetabular bearing surface. Thus, in selected patients with well-preserved surgical acetabular findings, the data from this review support considering hemiarthroplasty as a reasonable option to consider and discuss with patients who are willing to accept some thigh and groin pain in lieu of potentially avoiding other major problems of total hip arthroplasty in this challenging patient population.

## Figures and Tables

**Table 1:** Demographic comparison.

	Hemiarthroplasty *N* = 61	THA *N* = 182	*P* values
Avg. age (range)	56 (24–90)	59 (24–86)	0.219739
Males/females	24 males 37 female (61%)	80 males 102 females (56%)	0.631524
Pre-op HHS avg. (range)	67.2 (58–87)	67.7 (25–87)	0.630454
Post-op HHS avg. (range)	98 (70–100)	99.3 (75–100)	0.08252

**Table 2:** Comparison of thigh and/or groin pain.

	Hemiarthroplasty *N* = 61	THA *N* = 182	Chi-square	*P* values
Groin pain	10 (16.4%)	10 (5.5%)	5.81	0.0159
Thigh pain	7 (11.5%)	4 (2.2%)	7.08	0.0078

**Table 3:** Radiographic assessment.

	Hemiarthroplasty *N* = 61	THA *N* = 182	*P* values
Polywear ≥ 2 mm	n/a	13 (7%)	n/a
Stem subsidence	2 (3.2%)	1 (0.5%)	0.1562
Calcar erosion	0	6 (3%)	0.20906
Cup osteolysis	0	1 (0.5%)	1
Cup protrusio/migration	0	2 (1.9%)	1

**Table 4:** Complications.

	Hemiarthroplasty *N* = 61	THA *N* = 182	*P* values
Heterotopic ossification	1	5	1
Hematoma requiring surgery	1	3	1
Infection	0	3	0.574634
Dislocation	0	13	0.042452
VTE (PE or DVT)	0	0	1
Loose stems	2	1	0.156242
Cup migration/loosening	0	2	1
Premature polyethylene wear	0	1	1
Acute post-op sciatica	0	1	1

Total complications	4	29	0.083049

**Table 5:** Reasons for revision surgery.

	Hemiarthroplasty *N* = 61	THA *N* = 182	*P* values
Instability	0	8 (4.4%)	0.120797
Loose cup	0	2 (1.9%)	1
Loose stem	2 (3.3%)	1 (0.5%)	0.156242
Infection	0	3 (1.6%)	0.574634
Accelerated polywear	0	1 (0.5%)	1

Total revisions	2 (3.2%)	15 (8.2%)	0.252823
